# Kimchi and Other Widely Consumed Traditional Fermented Foods of Korea: A Review

**DOI:** 10.3389/fmicb.2016.01493

**Published:** 2016-09-28

**Authors:** Jayanta Kumar Patra, Gitishree Das, Spiros Paramithiotis, Han-Seung Shin

**Affiliations:** ^1^Research Institute of Biotechnology and Medical Converged Science, Dongguk University-SeoulGoyang, South Korea; ^2^Department of Food Science and Human Nutrition, Agricultural University of AthensAthens, Greece; ^3^Department of Food Science and Biotechnology, Dongguk University-SeoulGoyang, South Korea

**Keywords:** chongkukjang, doenjang, ganjang, gochujang, kimchi, fermentation, lactobacillus

## Abstract

Different types of fermented foods such as chongkukjang, doenjang, ganjang, gochujang, and kimchi are plentifully available and widely consumed in north eastern Asian countries including Korea. Among them, kimchi is one of the most popular Korean traditional food. It is prepared by fermenting the baechu cabbage together with other vegetables and lactic acid bacteria (LAB) with functional potential. Many types of ingredients are added to kimchi to enhance its taste, flavor, nutritional value, texture etc. A number of bacteria are involved in the fermentation of kimchi, but LAB are the dominant species in the fermentation process. The addition of other sub ingredients and formation of different by-products during fermentation eventually leads to eradication of putrefactive and pathogenic bacteria, and also increase the functionalities, nutritional and nutraceutical potential of kimchi. Kimchi possesses anti-inflammatory, antibacterial, antioxidant, anticancer, antiobesity, probiotic properties, cholesterol reduction, and antiaging properties. In the present review an attempt has been made to review the different types of fermented foods found in the Korean peninsula with detailed scientific research regarding preparation, processing, structure of the microecosystem, and health benefits of kimchi.

## Introduction

The unique geographical location of Korea and the isolation from neighboring countries imposed by rugged mountains from the north and rocky ocean from the east, south, and west, largely contributed to the development of a distinct ethnic group with unique culture. Through time, simplicity has become a basic notion of the Korean philosophy. This simplicity is also reflected in the food habits. A fundamental aspect of this culture has been the preservation of fish, meat, pulses, and vegetables from times of abundance to times of scarcity through lactic acid fermentation; a process applied for more than 1500 years (Han et al., 1998; Surh et al., 2008; Oh et al., 2014).

Intake of a specific dose of fruits and vegetables in the daily food to prevent different types of chronic pathologies and diseases such as coronary heart problems, hypertension, and risk of strokes has been recommended by the Food and Agriculture Organization (FAO) and the World Health Organization (WHO) (Swain et al., 2014). Consumers are always concerned about the safety of the types of food they consumed along with their nutritional and beneficial effect to the health (Endrizzi et al., 2009). Keeping this concept in mind, the preservation and storage of various food materials in order to increase their nutritional content and protect their shelf-life has been practiced since time immemorial and this process has been most commonly known as fermentation in most scientific terminology. This process has been developed in order to preserve different types of fruits and vegetables by organic acid and alcohols during their harvesting season and use them at the time of scarcity. Fermented foods and beverages, whether of plant or animal origin, play a vital role in the diet of people in many parts of the world including the Asian and the Western countries. Fermented foods not only provide important sources of nutrients but have also great potential in maintaining health and preventing diseases along with the addition of desirable flavor, texture, reduction of toxicity, and decrease in cooking time (Rolle and Satin, 2002; Kabak and Dobson, 2011).

Generally, the fermentation process is the slow breakdown of organic substances that is prompted by a group of microorganisms or enzymes and results in the alteration of carbohydrates to organic acids or alcohols (FAO, 1998). The fermentation products vary considerably due to the use of various raw materials and preparation techniques (Surh et al., 2008). Customarily the lactic acid (LA) fermentation of vegetables and fruits is a common practice for improvement of nutritional and sensory features of food products (Demir et al., 2006; Di Cagno et al., 2013). A number of lactic acid bacteria (LAB) including *Lactobacillus brevis, Lb. fermentum, Lb. plantarum, Leuconostoc mesenteroides, Weissella confusa* and *Pediococcus pentosaceus* are regularly retrieved and have been widely used in the fermentation process (Jung et al., 2014). A number of fermented food products including cereal-based fermented food and non-alcoholic beverages, fermented milks, fermented fruits and vegetables and fermented meat products etc. have been consumed in most parts of the world (Kabak and Dobson, 2011). However, there are some differences in the preparation of traditional foods and beverages from region to region. The process of fermentation of fruits and vegetables has been passed down to subsequent generations as a family tradition and techniques and thus there is no strict procedure of fermentation (Swain et al., 2014). However, drying and salting are the most common practices that are applied in the old food preservation procedure. A number of fermented foods available in the Korean cuisine have been studied to some extend (Jang et al., 2011; Dharaneedharan and Heo, 2016; Koo et al., 2016; Tamang et al., 2016). Among them, kimchi, chongkukjang, doenjang, ganjang, and gochujang may be regarded as characteristic ones. Moreover, kimchi has met worldwide recognition and commercial significance. In the present review, all available literature on the afore mentioned Korean fermented foods with particular focus on scientific research regarding preparation, processing, structure of the microecosystem and health benefits of kimchi are integrated and critically reviewed.

## Fermented food of Korea

There are numerous fermented foods and beverages, which are the essential element of the Korean cuisine and are consumed by the Koreans as well as many people around the world throughout the year. These fermented food products have also become popular in the western countries and are well appreciated by the people around the world. The major fermented food items, except the alcoholic beverages that are consumed nowadays in Korea, are basically divided into three broad categories (Table 1) (Surh et al., 2008).

**Table 1:** **Different types of fermented food available in the Korean peninsula and their medicinal values**.

**Category**	**Name**	**Preparation process**	**Photo**	**Medicinal value**	**References**
First category	Chongkukjang	Short term fermentation of boiled soybean seeds using *Bacillus subtilis* and rice straw	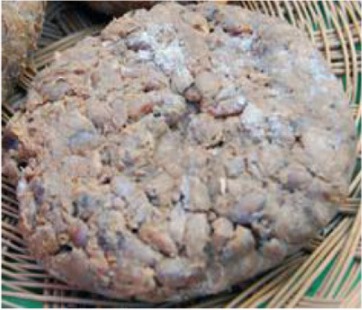	Rich in proteins, vitamins and minerals. Immunostimulant, antimicrobial, anti-inflammatory, antioxidant, neuro-protective etc.	Kang et al., 1998; Cho et al., 2000; Yang et al., 2003; Kim et al., 2004
	Doenjang	Fermentation of cooked soybean seeds with natural occurring bacteria	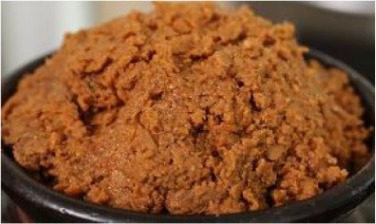	Anticancer, antimutagenicity, antioxidative, and fibrinolytic activity	Lim et al., 1999, 2004; Ra et al., 2004
	Ganjang	Soybean sauce prepared from fermented soybean	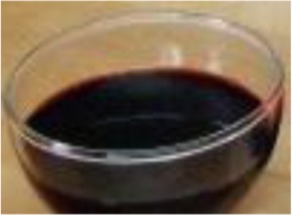	Antioxidant potential	Choi et al., 1990
	Gochujang	Fermented paste of red chili powder	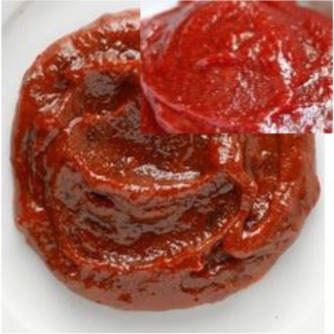	Antioxidant	Choi, 2012
Second category	Fish and Shellfish products	Fermented fresh fish and shellfish	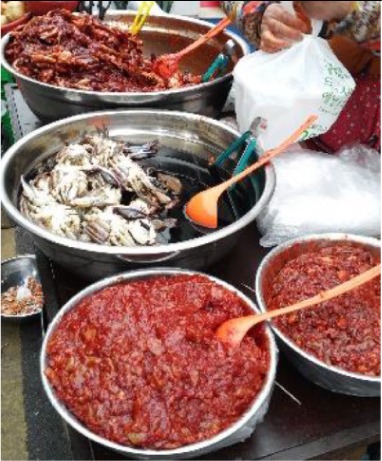	Essential source of vitamins, proteins, and minerals	Prester, 2011
Third category	Kimchi	Short fermentation of napa cabbage together with other ingredients	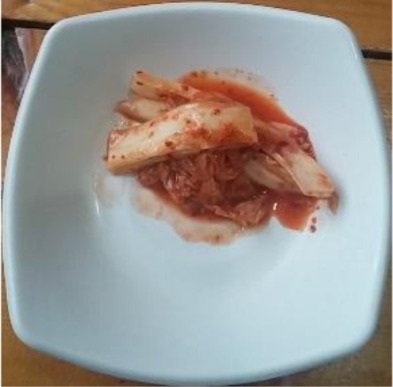	Antioxidant, anti-obesity, anticancer, antibacterial, cholesterol lowering, immune potential, source of functional foods	Kim et al., 1997; Wu et al., 2000; Yoon et al., 2004; Han et al., 2011; Park et al., 2014

### First category

The first category consists of the soy-based products, that includes chongkukjang (quick fermented soybean paste), doenjang (soybean paste), ganjang (soy sauce), and gochujang (hot pepper-soybean paste) (Surh et al., 2008). Traditionally, these types of fermented products are prepared once in a year, stored in large clay pots and consumed throughout the year.

#### Chongkukjang

Chongkukjang is a fermented product manufactured by short term fermentation of boiled soybean seeds using *Bacillus subtilis* in rice straw. It is one of the favorite traditional foods in Korea (Su et al., 2007; Kwon et al., 2011; Shin and Jeong, 2015). It contains a number of useful microorganisms and bioactive compounds that are absent from unfermented soybean products. Chongkukjang has the shortest fermentation period of 2–4 days and is fermented at a high temperature (40–43°C). The soybean proteins are degraded during fermentation process by the protein degrading enzymes of *B. subtilis*, and flavonoid glycosides are converted into aglycones by hydrolysis during fermentation, resulting in production of free amino acids along with related peptides (Nakajima et al., 2005; Kim N. Y. et al., 2008; Wei et al., 2015). The Koreans have been consuming Chongkukjang for hundreds of years. Significant amount of data suggests that Chongkukjang contains a number of proteins and minerals that can stimulate the generation and growth of human cells and strengthen the immune system (Choi et al., 2014; Shin and Jeong, 2015). Moreover, there are several reports on the bioactive potential of Chongkukjang such as antidiabetic, antiinflammatory, antimicrobial, antioxidant, blood pressure lowering activities, and neuroprotective effects (Kang et al., 1998; Cho et al., 2000; Yang et al., 2003; Kim et al., 2004; Kim N. Y. et al., 2008; Wei et al., 2015). Compounds like isoflavones (daidzein and genistein) are found at high concentrations in Chongkukjang and they were reported to possess the protective effect against oxidative damage related with atherosclerosis and cancer (Barnes et al., 1996; Anthony et al., 1998).

#### Doenjang

Doenjang is traditionally used in Korea as a basic seasoning (Kim et al., 2009; Nam et al., 2015). It is produced by the fermentation of cooked and crushed soybean seeds or blocks, *Meju*, by naturally occurring bacteria and fungi with brine in a container such as a porcelain pot and has been consumed for centuries as a source of protein and flavoring ingredient in Korea (Kim et al., 2009; Kwon et al., 2010). During recent times, the doenjang has been prepared commercially by various local firms around the Korean peninsula using a slightly modified procedure that varies in the quality as affected by the fermentation process, microbiota involved, and by the basic ingredients used, such as soybeans or a combination of soybeans and grains (Yoo et al., 2000; Park et al., 2002). Doenjang has attracted much attention due to its health-related beneficial properties such as anticancer, anti-mutagenicity, antioxidative, and fibrinolytic activity (Lim et al., 1999, 2004; Ra et al., 2004). Various types of microorganisms are involved in the fermentation of doenjang; the unique flavors and tastes are due to the decomposed products of soybean proteins from the soybean seed by the action of microorganisms during the fermentation process. A number of reports have stated that *B. subtilis* and *B. licheniformis* are the dominant microorganisms in doenjang along with *Aspergillus, Mucor*, and *Rhizopus* species (Kang et al., 2000; Nam et al., 2015). During the fermentation process, cleavage of β-glycosyl bond of isoflavone glucoside increases the content of isoflavone aglycones, including daidzein and genistein due to the rapid microbial growth (Nam et al., 2015). Daily intake of doenjang has been reported to suppress the body weight gain, cytokine levels, and serum oxidative stress in high-fat-fed mice (Nam et al., 2015). Similarly, the anti-inflammatory and anti-oxidative stress effects of doenjang have also been reported in the adipose tissue (Nam et al., 2015).

#### Ganjang

Ganjang is a kind of Korean soybean sauce made from fermented soybeans (Hong-beum, 2004). It contains approximately 15–20% salt, 50–70% water, free sugars, isoflavones, peptides, and organic acids that are produced from the soybeans during the fermentation process (Jeon et al., 2002; Shim et al., 2008). The sauce has a characteristic black color due to the presence of melanoidins, which are formed when carbonyl compounds and amino compounds combine together (Kim et al., 2011). The melanoidins present in soybean sauce are responsible for its antioxidant potential (Choi et al., 1990). Ganjang is prepared from the soybeans blocks, *meju*, which is dried for about 1 week and then tied with straw and dried for another 40 days. After the *meju* have dried, they are then fermented in a specially made clay pot mixed with salt and water. When the fermentation is complete, dark liquid separates, which is called ganjang (soy sauce or soya sauce) (Hong-beum, 2004).

#### Gochujang

Gochujang is a fermented paste made of red chili powder, glutinous rice powder, pureed soybeens and salt, seasonings like garlic and onion, sweetened with a little sugar syrup and fermented for long period in specially designed earthen vessels (Choi, 2012). It is an essential part of the Korean cuisine and is used in almost all the Korean foods like bibimbap, noodles etc. It is a basic ingredient for other sauses and pastes, it is mixed with the doenjang to make samjang, it is used to prepare the chogochujang, salad dressing etc. (Choi, 2012).

### Second category

The second type of fermented food that is popularly consumed in Korea is prepared from fish and shellfish. These products are consumed as such or are combined with kimchi (Surh et al., 2008). Fish, shellfish and their products provide a healthy source of essential vitamins, high quality proteins, minerals, and polyunsaturated fatty acids (Prester, 2011).

### Third category

The third category is the kimchi, which is most widely and popularly consumed not only in Korean peninsula but around the world. It is a major Korean traditional fermented food. Kimchi is prepared from the Chinese cabbage (*Brassica rapa* L. spp. *pekinensis* [Lour.] Han) and/or radish as its main ingredient, along with different kind of vegetables (Surh et al., 2008). The fermentation process is completed within short period of time. A detailed study on the preparation, processing, and microecosystem is discussed subsequently in the present review.

## “Kimchi” Korean well known fermented food

**Figure 1:**
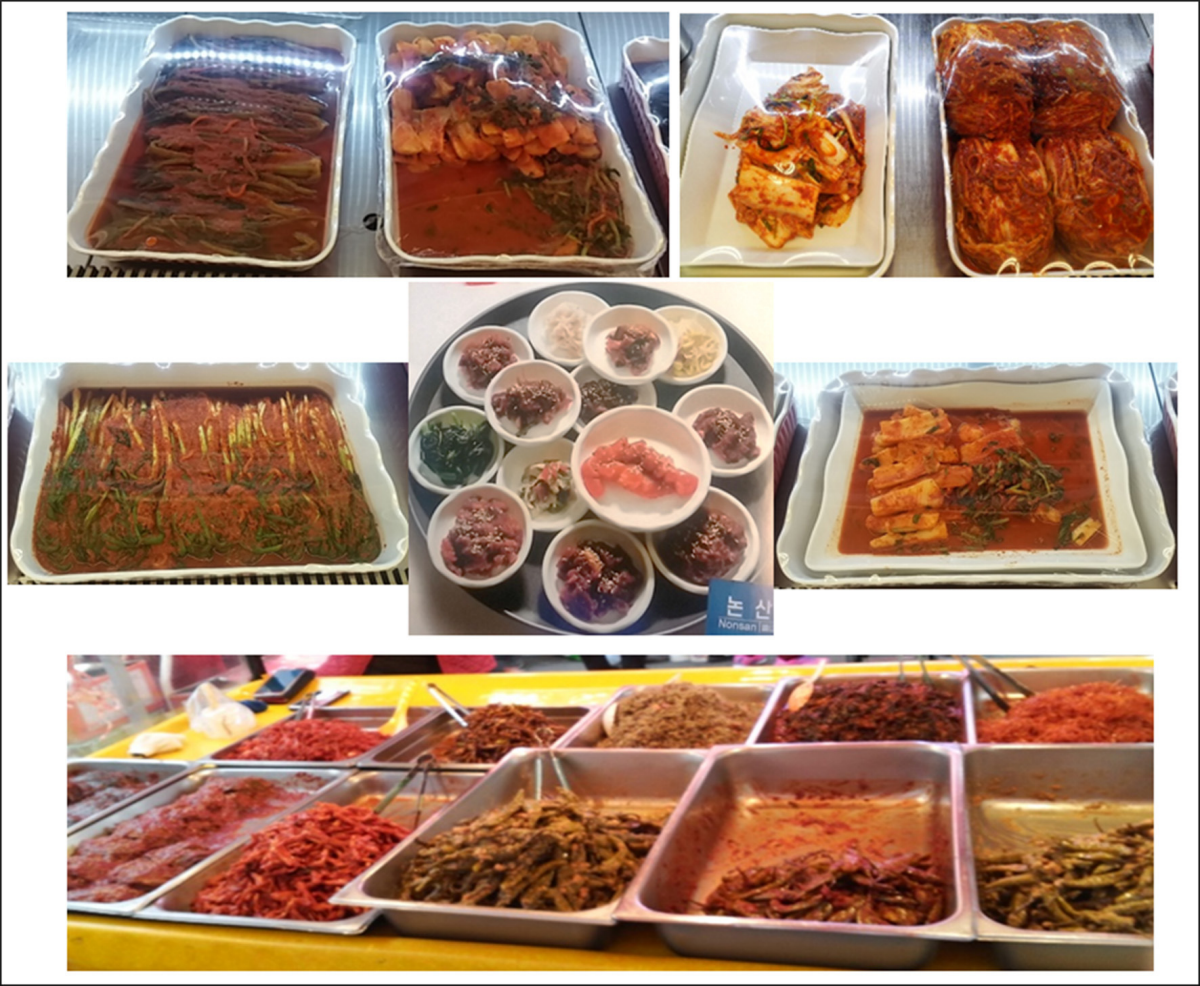
**Different types of kimchi available in Korean peninsula**.

Kimchi is the most important traditional fermented food in Korea and one of the most widely consumed in other East Asian countries like Japan and China. Information about kimchi can be retrieved from the ancient Korean book “*Samkuksaki*,” published in 1145 A.D., as well as in many other documents such as the subsequent “*Naehun*,” “*Hunmongjahoe*,” “*Sinjeung*-*yuhap*,” and “*Kanibuckonbang*” (Cheigh and Park, 1994; Jang et al., 2015; Yang H. J. et al., 2015). According to them, kimchi was considered as the outcome of a simple vegetable in brine fermentation prepared in a stone jar (Cheigh and Park, 1994). Since then, several kimchi types have been recorded according to variations in their composition or preparation method (Surh et al., 2008). In that sense, kimchi prepared with the use of leaf mustard, sweet potato, radish or young radish with leaves (*Dongchimi, Chonggak, Beeneul*), dropwort, various wild grasses, lettuce (*Gotchorri*), cucumber (*Sobagi, Ggagduki*), eggplant, pumpkin, burdock, sliced vegetables (*Nabak*), leek, scallion, garlic, chicken, pheasant, ear shell, green laver as well as seafood are available in local markets of the Korean peninsula (Figure 1).

### Kimchi ingredients and preparation

The wide variety of raw materials used for kimchi preparation makes it unique among the products for the production of which lactic acid fermentation is employed. This variety generates an array of products with escalating organoleptic intensity, which makes it suitable for every age and taste.

## Basic ingredients

The raw materials are divided into four classes (Cheigh and Park, 1994): (a) major raw materials, (b) spices, (c) seasonings, and (d) other additional materials. Although Chinese cabbage is more often reported as the major ingredient, as many as 30 different types of vegetables, including radish, young Oriental radish, ponytail radish, and cucumber may be used as well (Kim et al., 2012). The spices regularly used include red and black pepper, cinnamon, garlic, ginger, onion, and mustard. The seasonings most frequently used include salt and salt-pickled seafood, corn syrup, sesame seed, and soybean sauce. Finally, mushrooms as well as vegetables such as carrot, leek, and water cress, seafood like oyster and shrimp, cereals like barley and rice, fruits like apple and pear, meats like pork and beef and many more depending upon availability, geographical region and desired taste fall into the last class of ingredients. Qualitative and quantitative variation in the aforementioned ingredients is reflected in the sensorial properties of the final product; in that sense, adjustment of the taste is feasible. The Chinese cabbage kimchi, locally referred to as “Baechu,” is the most popular type of kimchi in Korea. For the preparation of this product the average composition of the different raw materials is as follows: Chinese cabbage 74–90%, radish 2.8–13.5%, garlic 1.4–2.0%, ginger 0.5–1.0%, onion 1.5–2.0%, green onion 1.0–3.5%, red pepper 1.8–3.0%, and a wealth of optional ingredients such as leek, shrimp and anchovy paste etc. each added below 2.0%; the final salt level is calculated at 2.5% (Park et al., 1994; Cho, 1999; Park and Cheigh, 2003; Lee et al., 2005; Cho et al., 2006).

## Preparation procedure

### Homemade kimchi

**Figure 2:**
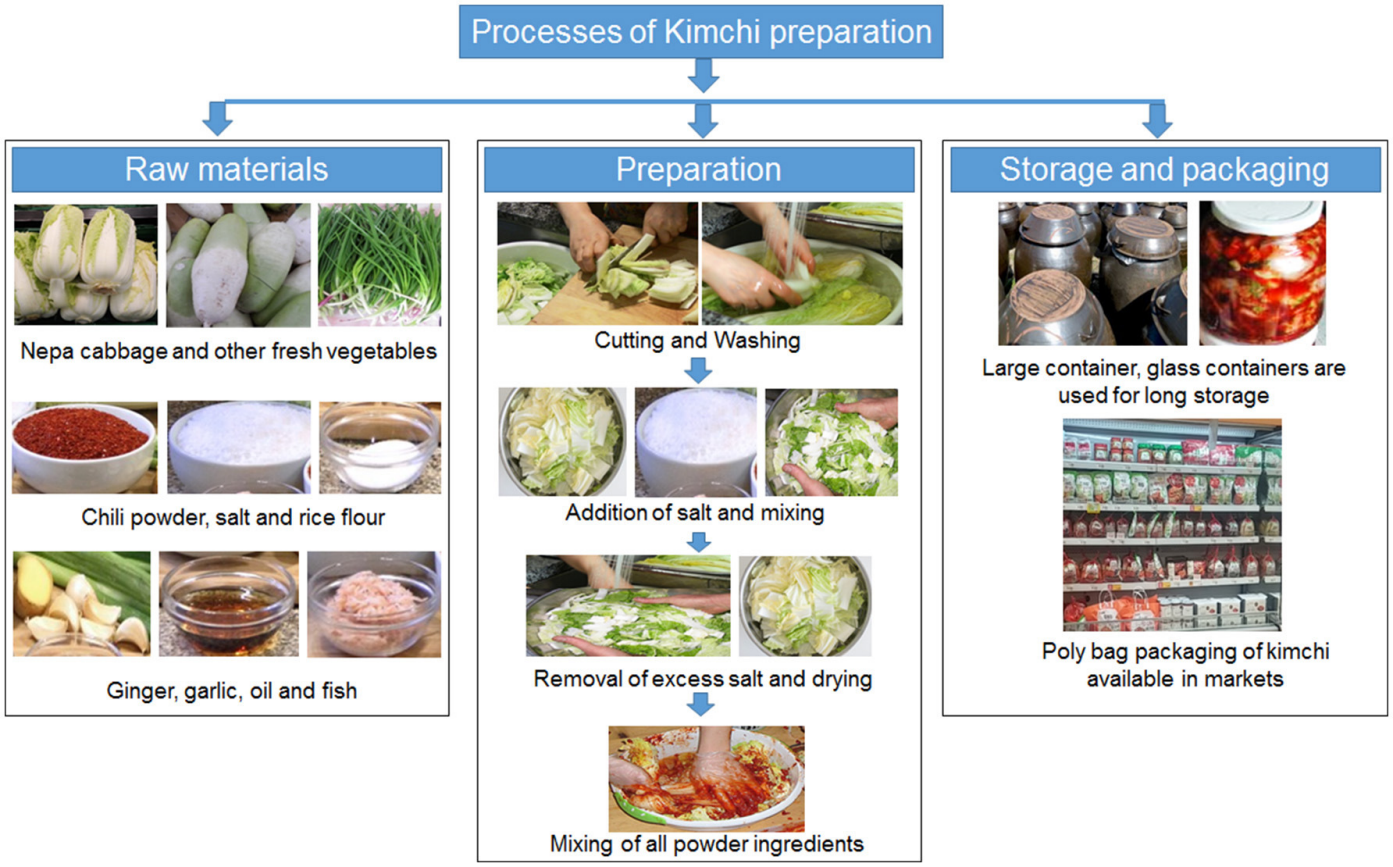
**A schematic representation of the traditional process for kimchi preparation**.

In Figure 2, the basic preparation method is described. Initially, all raw materials are collected. Their selection depends upon taste preferences, availability of raw materials, family tradition, social status etc. Chinese cabbage, the major ingredient, is trimmed to small pieces and thoroughly washed. The excess water is drained and brining takes place. During brining, a small amount of table salt is added and left for 2–3 h. During this time, washing, grading, cutting and mixing of the remaining raw materials takes place. When brining is completed, the excess amount of water is drained again and all the raw materials are mixed (Cheigh and Park, 1994; Park et al., 2014). Fermentation conditions depend upon consumption and storage needs; short-term consumption requires fermentation at room temperature whereas longer storage times requires fermentation at low temperature (5°C). The product can be called as kimchi only after the completion of the fermentation process (Kim et al., 2012; Oh et al., 2014).

## Commercial production of kimchi

Kimchi has become one of the most important globally popular food products, because of its taste and health claims; therefore its market has increased worldwide (Jung et al., 2014). The major challenge regarding industrial kimchi production is obtaining uniform quality. This task may be achieved by careful standardization of the whole production procedure, i.e., use of high quality raw materials, starter cultures and suitable fermentation conditions. Due to the large number of health claims, a large proportion of which is attributed to the microbiota itself (see paragraph “health benefits”), the criteria for the selection of starter cultures include the evaluation of the medicinal potential. In that sense, LAB such as *Ln. mesenteroides, Ln. citreum*, and *Lb. plantarum* have been successfully applied as starter cultures for kimchi preparation (Kim et al., 2012; Ryu et al., 2012). However, the adaptability to the kimchi microenvironment that may be assessed through persistence, as well as technological properties, such as production of organic acids, mannitol, compounds that may affect the product organoleptically, biogenic amines, vitamins, and bacteriocins should also be considered (Lee et al., 2011, 2015; Jung et al., 2014).

## Storage and preservation

The Korean made kimchi requires less quantity of salt due to the use of red pepper powder (Oh et al., 2014). As a result, kimchi can be stored for long periods of time facilitating commercialization that has increased sharply in countries like Korea, China, Japan, and the United States due to their popularity among the people (Kim et al., 2012). Kimchi is usually stored in two ways, either for 3 weeks at 4°C, which is considered as well ripened or for only 3–4 d at room temperature. The raw kimchi is eaten in various ways as salad mixed with sesame seeds, sesame seed oil and sugar, while the over-ripened kimchi is usually boiled with meat (*jigae*). It has been reported that the average consumption of kimchi for the Korean population on daily basis is 124.3 g, with maximum of 154.5 g consumed in the age group 30–49 years old (Surh et al., 2008). The most important problem in the commercialization of packaged kimchi is the continuous fermentation process by LAB during distribution and storage that eventually decreases significantly the quality of the product. Over-fermentation of kimchi results in excessive acidification (sour taste) due to the production of acid by LAB with softening of its texture and diffusion of color (Cheigh and Park, 1994; Lim et al., 2001). Thus, the extension of kimchi shelf life and maintaining of its quality by minimizing LAB growth, is a major concern for the kimchi industry (Swain et al., 2014).

## Biological changes during kimchi fermentation

### Physicochemical changes

Kimchi fermentation may be divided into four stages on the basis of the acidity produced (Codex, 2001): (1) initial stage with acidity <0.2%, (2) immature stage with acidity between 0.2 and 0.4%, (3) optimum-ripening stage with acidity between 0.4 and 0.9%, and (4) over-ripening or rancid stage with acidity >0.9%. The dominating LAB are responsible for the production of the acidity, through fermentation of the available carbohydrates. Type and quality of the raw materials, as well as fermentation conditions decisively affect the course of fermentation. Given the extended variety of the former and the variations observed in the latter, generalizations regarding microbial succession and dominance at species level may be at least uncertain, as will be discussed in the next paragraph.

### Microbial community structure

The involvement of different types of microorganisms in kimchi fermentation process was studied for the first time in Korea in 1939, and since then many LAB have been isolated and thoroughly characterized (Lee et al., 1997). Apart from this, many other traditional fermented foods were also studied for their microbiota composition (Jang et al., 2011; Dharaneedharan and Heo, 2016; Koo et al., 2016; Tamang et al., 2016). However, in the present study, emphasis was given on the kimchi microbiota because it is the most consumed fermented food in Asia and other continents of the world. Assessment of the kimchi microecosystem was initially performed by culture-dependent methods. However, due to the inherent limitations, the number of studies using culture-independent approaches, such as Denaturing Gradient Gel Electrophoresis (DGGE) and pyro-sequencing has been increased over the last decade (Jung et al., 2013; Jeong et al., 2013a; Park et al., 2014). Although application of these techniques has not improved our knowledge regarding the composition of the microecosystem, both qualitatively and quantitatively, an enhancement of speed as well as in some cases reliability has been observed.

In Table 2 the LAB species that participate in the microbial consortium during kimchi fermentation is presented. Several species, such as *Ln. carnosum, Ln. mesenteroides, Ln. gelidum, Ln. lactis, W. confusa, Lb. plantarum*, and *Lb. sakei* seem to be stable elements of the microecosystem whereas species such as *Ln. citreum, Ln. gasicomitatum, Ln. kimchi, W. koreensis, W. cibaria, Lb. spicheri, Lb. parabrevis, Lb. brevis*, and *Lb. curvatus* may be sporadically present (Kim and Chun, 2005; Lee D. et al., 2008). The factors affecting the course of fermentation as well as the dominating LAB population have been extensively studied. Incubation temperature has been recognized as the most important factor. Lee et al. (2005) applied PCR-DGGE to assess the composition of the LAB microbiota during fermentation at 10 and 20°C for 30 and 20 d, respectively. *W. confusa, Ln. citreum, Lb. sakei*, and *Lb. curvatus* formed the LAB consortium that prevailed from the beginning of the fermentation in both temperatures. In the case of 20°C this consortium was enriched with *Lb. brevis* and *Lc. lactis* subsp *lactis*. Cho et al. (2006) studied the effect of a preliminary incubation at 10 and 15°C for 4 and 2 d, respectively, before the main incubation that took place at −1°C for 90 days, on the composition of the microecosystem. *W. koreensis* was reported as predominant in both cases. Moreover, when pre-incubation took place at 10°C, *W. koreensis* dominated the fermentation from the beginning. On the contrary, *Ln. citreum* and *Ln. gasicomitatum* prevailed during preliminary incubation at 15°C. The latter species remained detectable until the end of fermentation. PCR-DGGE was also used by Hong et al. (2013) to compare the bacterial community changes during kimchi fermentation at 4 and 10°C. In the former case, *W. confusa, Lb. sakei, Lb. curvatus, Ln. gelidum*, and *Ln. carnosus* were reported to dominate the whole fermentation whereas in the latter case a consortium consisting of *W. confusa* and *Lb. curvatus* as stable elements throughout fermentation and occasionally by *Ln. citreum, Lb. parabrevis, Lb. sakei, W. koreensis*, and *Ln. mesenteroides* was reported.

**Table 2:** **Effect of composition and fermentation conditions on the structure of the lactic acid bacteria micro-community developed during spontaneous kimchi fermentation**.

**Kimchi composition—fermentation conditions**	**Microbiota**	**Method**	**References**
Chinese cabbage (74.5%), radish (13.5%), garlic (2.0%), ginger (0.5%), onion (2.0%), green onion (1.0%), red pepper powder (3.0%), leek (0.5%), shrimp paste (1.5%), anchovy paste (0.5%), sucrose (1.0%). a. 10°C for 4 d, then reduction to −1°C over 12 h b. 15°C for 2 d, then reduction to −1°C over 24 h	*Ln. carnosum, Ln. citreum, Ln. gasicomitatum, Ln. gelidum, Ln. kimchii, Ln. lactis, Ln. mesenteroides, Ln. inhae*^a^, *W. cibaria, W. confusa*^a^, *W. koreensis, Lb. curvatus*^a^, *Lb. pentosus, Lb. plantarum, Lb. sakei*	16S-rRNA gene restriction analysis, 16S-rRNA gene sequencing, specific PCR, DNA-DNA hybridization	Cho et al., 2006
Salted cabbage and kimchi ingredient mix a. 4°C for 30 d b. 10°C for 30 d	*W. confusa, W. koreensis*^c^, *Ln. citreum, Ln. mesenteroides, Ln*. *gelidum, Ln. lactis*^b^, *Ln. carnosum, Lb*. *parabrevis*^c^, *Lb. plantarum*^c^, *Lb*. *spicheri*^c^, *Ln. lactis*^c^, *S. salivarius, B. subtilis*	PCR-DGGE	Hong et al., 2013
Chinese cabbage 100 g, sugar 1 g, green onion 4 g, garlic 2 g, ginger 1 g, red pepper powder 2 g, and fermented anchovy sauce 1.4 g. a. 20°C for 20 d b. 10°C for 30 d	*W. confusa, Ln. citreum, Lb. brevis*^d^, *Lb. sakei, Lb. curvatus, Ln. lactis, Ln gelidum*^c^, *Se. marcescens*^c^	PCR-DGGE, 16S rRNA gene sequencing	Lee et al., 2005
Dongchimi Radish (3 kg), seasoning mixture (150 g) containing Korean leek, garlic, and ginger (6:3:1, w/w/w) and 4.5 l of 4.0% (w/v) solar salt water. Fermentation at 4°C for 100 days	*W. koreensis, Lb. plantarum, Lc. raffinolactis, Lc. piscium, Lc. lactis, Lb. pentosus, Lb. graminis, Ln. carnosum, Ln. kimchii, Ln. mesenteroides, Ln. inhae, W. soli, W. cibaria, Lb. sakei, Ln. gelidum, Ln. holzapfelii, Ln. lactis, Ln. gasicomitatum, Ln. citreum*	16S rRNA gene barcoded pyrosequencing	Jeong et al., 2013a

a *Only at 15°C*.

b *Only at 4°C*.

c *Only at 10°C*.

d *Only at 20°C*.

*B., Bacillus; Lb., Lactobacillus; Lc., Lactococcus; Ln., Leuconostoc; W., Weissella; Se., Serratia; S., Streptococcus*.

Sodium chloride is a basic ingredient of kimchi preparation. Reduction of sodium chloride generally in fermented products has been extensively studied due to the correlation with elevated blood pressure and increasing occurrence of cardiovascular diseases (Lee S. M. et al., 2012). In the case of kimchi, Song and Lee (2014) reported that its consumption could not be correlated to hypertension prevalence. The latter results from both sodium excess and potassium deficiency in the body; since vegetables are a major source of potassium, the high intake may neutralize the negative effects of sodium intake. Ahmadsah et al. (2015) reported that salt concentrations ranging from 1.0 to 2.1% had no effect on pH value, total titratable acidity, viable cell and coliform counts as well as the composition of the LAB microecosystem. The latter was reported to consist of *Lb. sakei, Ln. gelidum, Ln. lactis, Ln. mesenteroides, W. confusa*, and *W. soli* in all cases.

Occurrence of LAB in the raw materials used for kimchi preparation was also studied to some extent. Green onion was reported to contain *Weissella* spp. *Leuconostoc* spp. and *Lactococcus* spp. (Jung et al., 2012). Moreover, Kim et al. (2004) reported that green onion was the main source of *W. kimchi*. *Lactobacillus* spp., *Leuconostoc* spp., and *Weissella* spp. were reported as present in garlic samples (Jung et al., 2012). Interestingly, addition of garlic resulted in suppression of aerobic bacteria populations and enhancement of LAB population (Lee J. Y. et al., 2008). Finally, a significant delay of the fermentation process, especially the early period, upon addition of red pepper powder was reported by Jeong et al. (2013b) together with a higher proportion of *Weissella* spp. and a lower proportion of *Leuconostoc* spp. and *Lactobacillus* spp.

### Safety concerns

#### Selection of more suitable microorganisms for fermentation process

Kimchi has been in the epicenter of extensive criticism regarding both microbiological and chemical safety. This criticism has been intensified due to the recent outbreaks linked with Kimchi consumption; the first one took place in 2012 and was caused by enterotoxigenic *Escherichia coli* O169 while the second occurred in 2013 and was caused by norovirus GI.4 (Cho et al., 2014; Park et al., 2015). From a microbiological point of view, it has been widely accepted that lactic acid fermented foods are not a common vehicle of foodborne pathogens due to the antagonistic effect of the LAB; however, several studies as well as these outbreaks, have highlighted that such generalizations may be uncertain for reasons referring to both the raw materials and the adaptability of the pathogens (Burnett et al., 2000; Takeuchi and Frank, 2000, 2001; Beuchat, 2002; Klerks et al., 2007; Kroupitski et al., 2009, 2011; Mitra et al., 2009; Critzer and Doyle, 2010; Warriner and Namvar, 2010). Therefore, the ability of pathogenic bacteria to survive and proliferate during kimchi fermentation or storage has been studied to some extent.

Inatsu et al. (2004) assessed the survival of *E. coli* O157:H7, *Salmonella* Enteritidis, *Staphylococcus aureus*, and *Listeria monocytogenes* in both commercial and laboratory-prepared kimchi. Both types were inoculated with 5–6 log CFU/g of the pathogens and incubated at 10°C for 7 d. It was reported that all pathogens were able to survive. Moreover, only upon prolongation of the incubation did the population of the pathogens decreased to the enumeration limit. More accurately, *S. aureus* reached enumeration limit after 12 d, whereas *S*. Enteritidis and *L. monocytogenes* after 16 d. On the contrary, *E. coli* O157:H7 population remained at high levels throughout the 24 days of the experiment. These results were taken into consideration by Kim Y. S. et al. (2008) who applied heat treatment (85°C for 15 min) or a neutralization treatment (pH 7) at days 0 or 3 of kimchi fermentation in order to study the effect on *Bacillus cereus, L. monocytogenes* and *S. aureus* inoculated at 4–5 log CFU/g. Heat treatment at day 0 was more effective against *L. monocytogenes*, whereas at day 3 resulted in the complete inactivation of *L. monocytogenes*, significant population reduction of *B. cereus* but only marginal of *S. aureus*.

On the other hand, neutralization treatment at day 0 resulted in complete inactivation of *S. aureus* and significant decrease of *L. monocytogen*es population. However, upon neutralization treatment on day 3, complete inactivation of *L. monocytogenes* and significant decrease of *S. aureus* counts were observed. This issue was also assessed by Cho et al. (2011). In that study, the initial mixture of soongchimchae, i.e., a type of kimchi that combines fermented vegetables and meat, was spiked with 3–5 log CFU/g *E. coli* O157:H7 and *L. monocytogenes* and allowed to ferment at 4°C for 15 d. Both *E. coli* O157:H7 and *L. monocytogenes* counts gradually decreased during fermentation and were below detection limit after 14 and 15—post fermentation days, respectively.

#### Suitability of raw materials used in kimchi preparation

The high incidence of stomach cancer among Koreans raised some concerns regarding dietary habits. This high incidence was linked to the consumption of soybean paste due to aflatoxin content (Crane et al., 1970). This triggered a huge debate whether soybean paste as an ingredient and kimchi itself may be regarded as risk or protective factors (Kim et al., 1985; Messina et al., 1994; Lee et al., 1995; Kim H. J. et al., 2002; Kim J. H. et al., 2002; Nan et al., 2005; Wang et al., 2015).

From a food hygiene perspective, aflatoxin accumulation should be avoided by any possible means. Additionally, studies like the one by Park et al. (2003) that reported aflatoxin B1 and G1 gradual degradation to 80–90% after 2 months of Doenjang fermentation and 100% after 3 months of fermentation, as well as the potential of LAB to bind aflatoxins (Haskard et al., 2001; Peltonen et al., 2001; Lahtinen et al., 2004; Bueno et al., 2007; Hernandez-Mendoza et al., 2009) and concomitantly remove them from the medium, should be further exploited. Similarly, controversial debates related to the nitrates, nitrites, ethyl carbamate, secondary, and biogenic amines content of Kimchi are currently ongoing (Kim et al., 2000; Haskard et al., 2001; Peltonen et al., 2001; Lahtinen et al., 2004; Mah et al., 2004; Bueno et al., 2007; Hernandez-Mendoza et al., 2009).

### Health benefits of kimchi

#### Enhanced nutritional value

**Figure 3:**
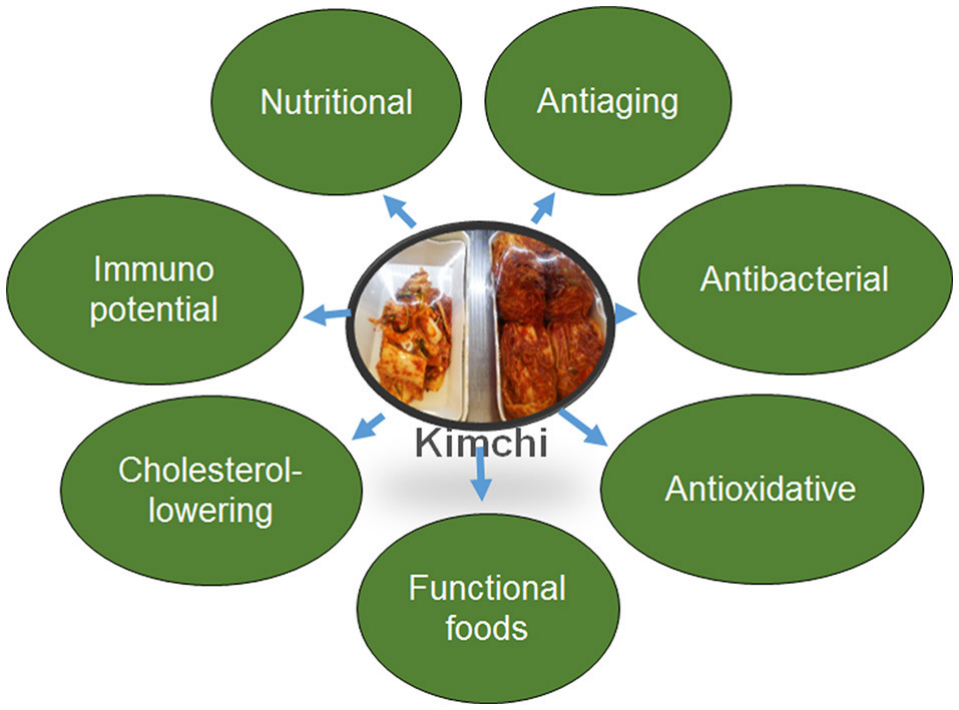
**Nutraceutical potential of Kimchi**.

Kimchi is recognized worldwide for the number of health claims that have been made (Figure 3). It was presented in Health Magazine in 2006 as one of the world's five healthiest foods (Lee G. I. et al., 2012; Park et al., 2014; Dharaneedharan and Heo, 2016; Tamang et al., 2016). These health benefits result from the utilization of raw materials of high nutritive value and the microbiota prevailing the fermentation. *Brassicaceae* vegetables have been reported to contain a number of compounds with health-promoting potential, including dietary fibers, minerals, amino acids, vitamins, carotenoids, glucosinolates, and polyphenols.

The nutritive content of Chinese cabbage [*Brassica rapa* L. *pekinensis* (Lour.) Olsson] in particular has been extensively studied; presence of vitamins A and C, 34 amino acids with threonine, arginine, γ-aminobutyric acid, alanine, asparagine, serine and glutamic acid being quantitatively the most important, 10 mineral elements among which Ca, Mg, K, Na in higher relative abundance, lutein and β-carotene as the major carotenoids has been reported with the variations in the presented values being assigned to genetic diversity, agricultural practices, harvesting stage, tissue as well as determination method (Wills and Rangga, 1996; Singh et al., 2007; Watanabe et al., 2011; Kim et al., 2014; Bhandari et al., 2015).

Therefore, the raw materials used in the preparation of kimchi result in the increased nutritional value, i.e., the high levels of vitamins such as vitamin C, b-carotene, vitamin B complex, etc., minerals such as Na, Ca, K, Fe, and P, dietary fiber, and other various functional components such as allyl compounds, gingerol, capsaicin, isothiocyanate, and chlorophyll.

#### Rich in a number of bioactive compounds

The phytochemicals such as indole compounds, b-sitosterol, benzyl isothiocyanate, and thiocyanate are the important active compounds found in kimchi, which have been reported to possess various medicinal potentials such as antiobesity, anticancer, antioxidant, and anti-atherosclerotic properties (Park and Rhee, 2005; Ahn, 2007; Park et al., 2014). In recent years, research has been focused on the polyphenolic and glucosinolate content due to the accompanying health claims that include protective effects against various types of cancer, cardiovascular and neurodegenerative diseases (van Poppel et al., 1999; Cohen et al., 2000; Kil, 2004; Stoclet et al., 2004; Scalbert et al., 2005; Kim et al., 2007; Singh et al., 2008; Han et al., 2011; Albarracin et al., 2012; Del Rio et al., 2013; Rodríguez-Mateos et al., 2014).

In Tables 3, 4 the polyphenolic and glucosinolate content of Chinese cabbage are presented, respectively. Regarding the former, sinapic acid has been reported as the major phenolic compound whereas gluconapin, progoitrin and glucobrassicin have been reported as the major glucosinolate (Mattila and Hellstrom, 2007; Chen et al., 2008; Kim et al., 2010; Cartea et al., 2011; Hong et al., 2011; Lee et al., 2014; Seong et al., 2016). In both cases the fluctuation presented is due to the analysis of different edible parts, varieties, or cultivars.

**Table 3:** **Minimum and maximum amount (mg/100 g dry weight) of polyphenolics present in Chinese cabbage**.

**Polyphenols**	**Seong et al., 2016**	**Mattila and Hellstrom, 2007**
Caffeic acid	nd-1.39 (0.06)	0.54 (0.046)
Sinapic acid	6.01 (0.43)–8.00 (0.08)	5.2 (0.44)
*p*-Coumaric acid	2.20 (0.07)–2.89 (0.17)	0.42 (0.035)
Ferulic acid	nd-0.47 (0.05)	1.4 (0.12)
Myricetin	0.80 (0.00)–0.83 (0.01)	
Vanillic acid		0.13 (0.015)

*Standard deviation is given in parenthesis; nd: not detected*.

**Table 4:** **Mimimum and maximum amounts of glucosinolates present in Chinese cabbage**.

	**(Chen et al., 2008) (mg/100 g FW)**	**(Kim et al., 2010) (umol/g DW)**	**(Lee et al., 2014) (umol/g DW)**	**(Hong et al., 2011) (umol/g DW)**
**ALIPHATIC GSs**				
Glucoalyssin	0.19 (0.02)–1.16 (0.34)	0.27 (0.00)–3.66 (0.65)	0.14–0.57	nd-0.45 (0.05)
Glucobrassicanapin	0.55 (0.15)–0.76 (0.13)	0.49 (0.34)–8.08 (6.89)	0.16–24.78	3.09 (0.15)–8.18 (0.39)
Glucoerucin	0.33 (0.08)–1.35 (0.21)	0.04 (0.02)–0.94 (0.88)		nd
Gluconapin	0.07 (0.02)–6.57 (1.52)	0.40 (0.06)–8.99 (0.52)	0.09–26.02	nd-1.53 (0.08)
Gluconapoleiferin		0.06 (0.02)–2.34 (2.75)		
Glucoraphanin	nd-0.40 (0.13)	0.03 (0.00)–0.49 (0.48)		nd
Progoitrin	1.44 (0.51)–4.93 (1.49)	0.23 (0.06)–3.97 (2.06)	0.55–4.33	2.49 (0.00)–5.31 (0.40)
Sinigrin	nd-0.44 (0.16)	0.04 (0.01)–0.15 (0.03)	0.09–1.96	
Total	2.79 (0.72)–15.42 (3.80)	1.94 (0.80)–19.71 (13.07)		
**INDOLIC GSs**				
4-hydroxyglucobrassicin	0.02 (0.00)–0.05 (0.01)	0.02 (0.00)–1.47 (0.84)	nd-0.32	0.40 (0.07)–1.48 (0.13)
4-methoxyglucobrassicin	0.78 (0.20)–2.62 (0.41)	2.07 (0.77)–4.83 (0.62)	0.21–4.35	4.94 (0.16)–6.08 (0.30)
Glucobrassicin	4.05 (1.18)–10.31 (1.72)	0.13 (0.04)–6.81 (1.72)	0.10–1.66	1.05 (0.09)–3.80 (0.08)
Neoglucobrassicin	0.53 (0.10)–5.49 (1.19)	0.01 (0.01)–0.55 (0.71)	nd-0.47	
Total	11.67 (2.02)–12.18 (2.66)	2.22 (0.59)–10.04 (2.16)		
**AROMATIC GSs**				
Gluconasturtiin	1.37 (0.34)–4.41 (0.99)		nd-2.61	
Glucoberteroin + aromatic GSs		0.31 (0.12)–3.31 (0.51)		

*Standard deviation is given in parenthesis. FW, Fresh Weight; DW, Dry Weight; nd, not detected*.

Apart from Chinese cabbage, radish, and onion are very often added to the mix, both of which are very important sources of dietary phytochemicals such as polyphenols, thiosulfates, and anthocyanins (Manach et al., 2004; Mattila and Hellstrom, 2007; Slimestad et al., 2007; Tedesco et al., 2015). Onion is a major source of flavonoids, particularly quercetin glucosides (Srinivasan, 2014) with reported anti-inflammatory (Gerates et al., 2007; Bureau et al., 2008; Lee et al., 2010; Endale et al., 2013; Yang D. et al., 2015) and anti-carcinogenic (Djuric et al., 2012) action.

Capsaicin and piperine are the major ingredients of red and black pepper, respectively. The antioxidant activity of both substances has been well documented (Pramanik et al., 2011; Arcaro et al., 2014). Garlic is rich in organosulfur compounds known for their antiinflammatory (Kumar et al., 2013; Schäfer and Kaschula, 2014) anticarcinogenic (Wang et al., 2012; Borkowska et al., 2013; Capasso, 2013) and cardioprotective potential (Stabler et al., 2012; Ried et al., 2013). Finally, a wide range of biological activities including anticancer (Ishiguro et al., 2007; Karna et al., 2012; Yang et al., 2012; Brahmbhatt et al., 2013) and antioxidant ones (Stoilova et al., 2007) have been assigned to ginger.

#### A good source of pharmaceutical perspectives

The potential for weight control in both mice and humans has been adequately documented. In the first case the antiobesity action that was accompanied by several other effects such as reduction of adipose tissue weights, adipocyte size, and inflammatory response in epididymal fat tissues, regulation of serum lipid profiles, insulin, eptin, and adiponectin levels, hepatic lipogenesis were assigned to the use of *Ln. mesenteroides* strain DRC 0211 (Cui et al., 2015) and *W. koreensis* strain OK1-6 (Park et al., 2012). The potential of *Ln. kimchi* strain GJ2 was described by Jo et al. (2015). In that study, a high-fat and high-cholesterol diet supplemented with kimchi made with the use of the aforementioned strain was fed to rats. A significant reduction of serum total cholesterol, triglyceride, low-density lipoprotein cholesterol levels, the atherogenic index, cardiac risk factor and triglyceride and total cholesterol levels in liver and epididymal adipose tissue were observed. Similar effects were observed by Kim E. K. et al. (2011).

Various researchers have indicated that consumption of kimchi on regular basis helps to decrease the level of cholesterol in the body (Kim and Lee, 1997; Kil et al., 2004). In the latter study, ingestion of fermented kimchi exhibited a series of positive effects on a variety of factors associated with metabolic syndrome, including systolic and diastolic blood pressures, percent body fat, fasting glucose, and total cholesterol, compared with the fresh kimchi suggesting that fermentation provides additional favorable effects for improving metabolic parameters. The red pepper powder used in kimchi is rich in capsaicin, which can cause loss of body fat by stimulating spinal nerves and activates the release of catecholamines in the adrenal glands of the body (Park et al., 2014). This compound increases the body metabolism and thus decreases the fat content (Yoon et al., 2004). Moreover, Kim E. K. et al. (2011) suggested that the consumption of fermented kimchi may affect obesity, lipid metabolism and inflammatory processes in human as well.

Furthermore, there are a number of health benefits with consumption of Kimchi as a part of the daily diet (Choi et al., 2015; Tamang et al., 2016). The antiaging potential of kimchi in the brain of senescence-accelerated mouse was reported by Kim J. H. et al. (2002) and using stress induced premature senescence of WI-38 human fibroblasts challenged with hydrogen peroxide was reported by Kim B. K. et al. (2011). Kimchi may have important role in delaying aging through the reduction of free radical production and increase in anti-oxidative enzyme activities. Similarly, kimchi also possess antibacterial potential that has been attributed to the presence of sulfur containing compounds and various LAB (Kil et al., 2004). The kimchi supplemented with Korean mistletoe extract had greater anticancer potential (80% inhibition ratio) than the kimchi without Korean mistletoe extract (62%) against the HT-29 human colon carcinoma cells (Kil, 2004). Likewise, different kinds of salt, natural sea salt (NS) without bittern or baked salt used to increase the taste of kimchi have been reported to increase the anticancer potential of kimchi as well (Han et al., 2009). Consumption of kimchi on a regular basis helps to increase the immune cell development and growth (Kim et al., 1997; Wu et al., 2000).

## Conclusions

A number of fermented food are being eaten by the people around Korean peninsula since time immemorial. These foods have become an essential part of the Korean food culture and tradition and are enriched with a number of nutritional and medicinal values. Among the fermented foods, kimchi is most widely eaten and adored throughout the world. Its main ingredient is the napa cabbage and red chili powder together with a number of vegetables and spices enriched with functional LAB. It has various nutritional and nutraceutical potential and its quality is enhanced by manipulating the different kinds and amounts of ingredients and fermentation conditions. Although there are a number of challenges in the kimchi processing and production, however this fermented food could play a major vital role in the global food industry. Thus, kimchi can serve as one of the best healthy foods available in the world and the information on its nutraceutical and nutritional potential could increase its use.

## Author contributions

JP and GD collected literature and wrote the review manuscript. JP, SP, and HS edited the manuscript. All the authors read and approved the manuscript.

### Conflict of interest statement

The authors declare that the research was conducted in the absence of any commercial or financial relationships that could be construed as a potential conflict of interest.
